# Molecular Aspects of Plant Salinity Stress and Tolerance

**DOI:** 10.3390/ijms22094918

**Published:** 2021-05-06

**Authors:** Jen-Tsung Chen, Ricardo Aroca, Daniela Romano

**Affiliations:** 1 Department of Life Sciences, National University of Kaohsiung, Kaohsiung 81148, Taiwan; 2 Department of Soil Microbiology and Symbiotic Systems, Estación Experimental del Zaidín (CSIC), 18008 Granada, Spain; ricardo.aroca@eez.csic.es; 3 Department of Agriculture, Food and Environment, University of Catania, 95123 Catania, CT, Italy; dromano@unict.it

Salinity is one of the major abiotic stresses that inhibit the growth, development, and productivity of crops, particularly in hot and dry areas of the world. It is an intensive topic on which many studies have been conducted, with the aim of understanding the physiological and molecular responses involved in plant salinity stress. In recent years, with the rapid progress of molecular technologies, scientists have acquired more advanced tools to reveal in-depth mechanisms and to establish crop breeding programs for enhancing plant salinity tolerance. This Special Issue, entitled “Molecular Aspects of Plant Salinity Stress and Tolerance”, collected 13 innovative publications which could enrich our knowledge about the molecular mechanisms of plant salinity stress.

It has been shown that investigations using advanced analyzed tools associated with multi-omics are reliable to gain new insights into the mechanisms associated with responses to salinity stress. Song et al. profiled the transcriptome together with the evaluation of photosynthetic efficiency of photosystem II and the amount of free amino acids in watermelon seedlings when exposed to short-term salinity stress [1]. They revealed that certain genes were found to express differentially in response to salinity, and these genes might code for transcription factors, or were proposed to be related to primary metabolism, endocytosis, hormonal pathways, and transporters. Isayenkov et al. investigated strategies for adaptation to salinity in seaside barley, using nuclear magnetic resonance imaging associated with multi-omics approaches [2]. The authors proposed that seaside barley has developed specific mechanisms involving the capability to regulate concentrations of Na^+^ and Cl^−^ in leaves and morphological and physiological adaptations of roots when subjected to salinity stress. 

For monitoring the status of the cell wall, plants have evolved a system, namely, cell wall integrity (CWI) [3]. Liu et al. provide a review on the roles of CWI in salinity tolerance and propose that the genetic engineering of CWI-related genes using genome editing technology might generate salt-tolerant varieties for applications in the future.

Jasmonates (JAs) are lipid-based plant hormones that regulate an array of processes in plants, particularly involved in defense mechanisms and stress tolerance. Delgado et al. refined the role of JAs in plant salinity tolerance, chiefly based on a genome-wide association study, and concluded that MYC2 transcription factor and JASMONATE ZIM-DOMAIN repressors are key components in JA signaling [4]. The authors provide a perspective that the knowledge of JAs against plant salinity stress might be applied as a guide in breeding programs.

Rice is one of the most important staple crops, as well as an intensively studied model for plant salinity stress and tolerance. Ponce et al. contributed a review focusing on the molecular mechanisms of salinity tolerance in rice [5]. The authors stated that the most investigated plant hormone in plant salinity stress is abscisic acid, and the recently identified mechanisms together with the key genes involved are critical to the breeding programs of highly salt-tolerant cultivars in the future.

Theoretically, biotechnological tools could be applied to identify candidate genes and subsequently alter the patterns of gene expression using the classical gene transfer method or targeted genome editing for acquiring salinity tolerance or inducing mutation to obtain salinity-tolerant genotypes. Previously, calmodulin-like proteins (CMLs) were found to be involved in salinity stress tolerance in Arabidopsis. Zhang et al. isolated a CML gene, *MpCML40*, from Pongamia, and found that its heterologous expression could improve salinity tolerance in yeast cells and enhance the rate of seed germination and the length of roots when exposed to salt and osmotic stresses in Arabidopsis [6]. The authors suggested that *MpCML40* contributed to the proline accumulation for reducing the damage caused by reactive oxygen species. A stress-induced aquaporin, *ZxPIP1;3*, was identified by Li et al. from succulent xerophyte, is belonging to the PIP1 subgroup of plasma membrane intrinsic proteins (PIPs) [7]. The overexpression of *ZxPIP1;3* in Arabidopsis showed certain improvements in growth and physiological attributes which lead to more efficient photosynthesis under salinity and drought stresses. Tran et al. tested the ion transport capacity of a PIP gene, *HvPIP2;8*, when expressed in the oocytes of *Xenopus laevis*. It was shown that the expression of *HvPIP2;8* enhanced the activity of water as well as Na^+^/K^+^ transport and might be involved in the responses when subjected to salinity stress in barley [8]. Kawakami et al. isolated one gene, *SvHKT1;1*, coded for a sodium transporter from a halophytic turf grass, *Sporobolus virginicus* [9]. The authors found that under severe salinity stress, SvHKT1;1 could prevent the excess accumulation of shoot Na^+^ in *S. virginicus*. Zhang et al. investigated the role of soybean transcription factor GmbZIP15 in regulating gene expression under abiotic stresses [10]. The resulting data showed hypersensitivity to abiotic stress in soybeans when overexpressing *GmbZIP15*, and thus they proposed that GmbZIP15 might be a negative regulator in plant salinity and drought stresses.

In agriculture, the application of genetic engineering to introduce genes for acquiring salinity tolerance has certain limitations. An alternative method to enhance plant salinity tolerance is using green inoculants of plant growth-promoting rhizobacteria (PGPR), such as salinity-tolerant (halophilic) plant-associated bacteria. Miller and Nielsen summarized the molecular mechanisms involving the interactions between plants and salinity-tolerant bacteria and proposed their potential applications in improving crop production under salinity stress [11]. In the review by Ha-Tran et al., the authors identified that PGPR is a promising agent for seed bio-priming to enhance seed vigor and germination and the uniformity of subsequent seedling growth under salinity stress [12]. The advantages of the PGPR together with the fluctuation of antioxidants and osmolytes in PGPR-treated plants were comprehensively discussed, and the authors suggested that further investigation on more complex interactions between plants and PGPR needs integrative multi-omics and systems biology, and other advanced tools.

Plant breeding programs for developing salinity-tolerant crops are crucial for improving their growth, yield, and product quality in salt-affected fields. In rice breeding, it has been recognized that salinity tolerance is contributed by multiple genes; consequently, it is a complex quantitative trait which is usually difficult to be evaluated. Qin et al. reviewed the advances in rice breeding for salinity tolerance, including the progress of molecular markers, genetic mapping, genetic engineering, and the interventions of biotechnology tools [13]. The authors suggested that the resulting high-salinity-tolerant germplasm is valuable for rice breeding programs in the future.

In conclusion, the advances in plant salinity stress and tolerance presented in this Special Issue include mechanistic insights revealed using powerful molecular tools together with multi-omics, and gene functions studied by genetic engineering and advanced biotechnological methods. Additionally, the use of plant growth-promoting rhizobacteria in the improvement of plant salinity tolerance and the underlying mechanisms and progress in breeding for salinity-tolerant rice are comprehensively discussed. Clearly, the published data have made significant progress in expanding our knowledge in the research field of plant salinity stress, and the full results are valuable in developing salinity-stress-tolerant crops, benefiting their quality and productivity, and eventually, supporting the sustainability of the food supply in the world.
